# New Inventions

**Published:** 1896-02

**Authors:** 


### NEW INVENTIONS.

A detachable horseshoe, made so as to be adjusted to a permanent shoe with recesses for calkings, and secured by lugs.

A horse-training apparatus, consisting of a rope around the upper jaw, with a knot over the front of the nose, and then through guides on headband; then passing to the hips and fastened to fixed part of the shafts. Raising the hind parts produces pressure on the nose. Kneebands attached by rope to the head-halter are also connected with rope running over the head.

